# The Enigmatic Schizoglyphid Mite *Oriboglyphus maorianus* gen. and sp. n. and Its Implications for Astigmatid Life Cycle Evolution †

**DOI:** 10.3390/life15071085

**Published:** 2025-07-10

**Authors:** Pavel B. Klimov, Vasiliy B. Kolesnikov, Matt Shaw, Qing-Hai Fan, Zhi-Qiang Zhang, Barry OConnor

**Affiliations:** 1 Lilly Hall of Life Sciences, Purdue University, G-225, 915 W State St., West Lafayette, IN 47907, USA; 2 Papanin Institute for Biology of Inland Waters, Russian Academy of Sciences, 152742 Borok, Russia; jukoman@yandex.ru; 3 Australian Museum, 1 William Street, Sydney, NSW 2010, Australia; matt.shaw@australian.museum; 4 Plant Health & Environment Laboratory, Ministry for Primary Industries, Auckland 1072, New Zealand; qinghai.fan@mpi.govt.nz; 5 School of Biological Sciences, The University of Auckland, Auckland 1010, New Zealand; zhangz@landcareresearch.co.nz; 6 Manaaki Whenua Landcare Research Group, Bioeconomy Science Institute, 231 Morrin Road, St Johns, Auckland 1010, New Zealand; 7 Museum of Zoology, University of Michigan, 3600 Varsity Drive, Ann Arbor, MI 48108, USA; bmoc@umich.edu

**Keywords:** astigmatid mites, phoretic deutonymph, phoretic tritonymph, new genus, life cycle, ontogeny, morphology, termites, New Zealand

## Abstract

A detailed morphological characterization is presented for *Oriboglyphus maorianus* gen. et sp. nov., a newly discovered modern representative of the relict family Schizoglyphidae, found in a nest of the termite *Stolotermes ruficeps* in New Zealand. This is the second extant schizoglyphid species known from modern material, and its discovery provides rare insight into a transitional stage in the evolution of astigmatid life cycles. The phoretic stage appears to be a tritonymph—rather than the typical deutonymph—based on key morphological traits including three-segmented palps, three pairs of genital papillae, and the presence of a pharynx. These features suggest that early astigmatid mites evolved multiple ontogenetic routes to dispersal, including tritonymphal, deutonymphal, and possibly adult phoresy, before the canalization of life cycles around deutonymphal dispersal. The persistence of putatively ancestral traits in schizoglyphids, along with their apparent ecological conservatism in termite nests, indicates the role of environmental stability in preserving modes of life history that otherwise appear extinct. We also provide a key to describe Schizoglyphidae species and discuss the implications of this discovery for understanding the origins of phoresy-related metamorphosis in Astigmata.

## 1. Introduction

Until recently, among astigmatid mites, the family Schizoglyphidae remained enigmatic, with only two known species. In 1978, Mahunka established a new family, Schizoglyphidae, to accommodate *Schizoglyphus biroi* Mahunka, 1978, a species collected in western New Guinea [1], though its true host associations remained dubious. More recently, another member of this family, *Plesioglyphus lebanotermi* Sendi et al. 2025, was discovered phoretic on the termite *Lebanotermes veltzae* Engel, Azar et Nel, 2011 in Early Cretaceous Lebanese amber, 129 Ma [2]. This record, along with a report of a modern schizoglyphid associated with *Stolotermes ruficeps* Brauer, 1865 in New Zealand from the same study, suggests that schizoglyphids have maintained their associations with termites from at least the Early Cretaceous to the present, representing the oldest unambiguous continuous phoretic association between mites and their arthropod hosts [2].

In Astigmata, the evolution of phoresy, involving both behavioral and morphological specialization, was a key adaptation that enabled dispersal via an enormous range of larger organisms (phoresy) allowing access to many patchy and ephemeral habitats [3,4,5]. Examples include fungal sporocarps, tree sap flows, dung, carrion, nests of vertebrates and invertebrates, phytotelmata, decaying wood, and other decomposing organic matter [6,7]. Phoresy enables mites to escape depleted food sources by attaching to a larger, more mobile organism that co-occurs in the same habitat and is likely to transport them to a similar new food resource [3,4,5,8,9]. Upon arrival, mites may continue their development for several generations until resources are exhausted, at which point a new phoretic cycle begins. Non-schizoglyphid Astigmata typically achieve phoresy as deutonymphs, a facultative (optional) stage inserted into the middle of their life cycle [7,10].

Across mites (Acariformes and Parasitiformes), the phoretic ontogenetic stage is highly conserved among unrelated lineages and restricted to either the deutonymph (immature males and females) or adults (usually inseminated females only, or both males and females). These two strategies differ in timing and reproductive trade-offs: (i) deutonymphs disperse early but delay reproduction on the new resource, potentially risking competition from faster-reproducing organisms, or (ii) adults disperse later but can reproduce immediately upon arrival at the new resource. Having both phoretic and reproductive functions in a single stage presents a significant challenge, as these functional roles may be mutually exclusive. For instance, the stage must balance the need for attachment structures, such as claws or suckers, with those required for reproduction, like a relatively large body cavity (to accommodate large volumes of eggs), specialized ovipositors, mating and sperm storage structures. These competing demands can limit the efficiency of each function, making it difficult to optimize both simultaneously. In line with this, phoretic adults typically exhibit limited adaptations for phoresy, whereas phoretic deutonymphs may be highly specialized, having an array of suckers and other attachment structures [10,11,12]. In Astigmata, they often lack functional mouthparts, as they do not feed orally, though non-oral feeding—unique to this stage—may still occur [13,14].

Until recently, the origin of the highly specialized phoretic deutonymph in Astigmata remained enigmatic, as no immediately similar forms were known from their oribatid mite ancestors [15]. However, paleontological evidence now suggests that their evolution was likely gradual rather than punctuated. This view is supported by the discovery of intermediate forms in the fossil record which bridge the morphological gap between ancestral and derived stages. For instance, fossil phoretic stages of *Levantoglyphus sidorchukae* Klimov et al., 2021 (the only species in family Levantoglyphidae) from the Cretaceous have a well-developed subcapitulum, chelicerae, and palps—structures that, although similar in form to those of their ancestors, exhibit modifications indicative of functional shifts. In particular, the palps appear to have become increasingly specialized for sensory roles, likely enhancing host-seeking behaviors [16]. These transitional forms point to stepwise morphological refinements of phoretic adaptations during early astigmatid evolution; however, the evolution of phoresy-related ontogenetic pathways still remains enigmatic in this lineage.

Phoretic schizoglyphid nymphs, exhibiting numerous plesiomorphic traits, may provide important insights into the early evolution of phoresy-related ontogenetic pathways in Astigmata. Notably, these nymphs exhibit a mix of morphological features typical of both heteromorphic deutonymphs—such as an attachment organ and a reduced gnathosoma adapted for host-seeking rather than feeding—and tritonymphs, including the presence of three pairs of genital papillae. A key unresolved question is whether their phoretic stage corresponds to either deutonymph or tritonymph [17]. Answering this question could clarify whether the early evolution of Astigmata was characterized by an initial burst of diversification [18,19,20,21,22,23], generating a variety of life cycles upon which natural selection acted, ultimately resulting in a single, predominant life-history strategy, as observed in non-schizoglyphid Astigmata. Early burst models suggest that high initial diversity arises due to low competition and abundant resources, i.e., the empty ecospace model [24,25,26,27]. Astigmata was likely the first mite lineage to exploit a combination of flexible developmental pathways—whereby a phoretic stage appears conditionally in response to environmental triggers, such as the depletion of ephemeral resources—and phoresy, where mites disperse on larger organisms and gain an adaptive advantage through faster life cycles upon arrival in a new habitat. As such, early Astigmata may have experienced low competition and abundant patchy resources in the initial stages of their evolution. It is possible that during this period, multiple life cycles and morphologies arose, but only a subset persisted into modern lineages, with many early forms going extinct. In other words, this hypothesis suggests that the initial radiation of life cycles was broad in Astigmata, followed by selection favoring a dominant, more successful developmental pathway (early burst model) [18,28,29].

During a brief collection trip to New Zealand in 2022, P.B.K. collected phoretic nymphs of an undescribed extant genus and species of Schizoglyphidae, *Oriboglyphus maorianus* gen. et sp. n., in association with the early-derivative native termite *Stolotermes ruficeps* (Kalotermitidae). While this mite species was briefly discussed in relation to the fossil schizoglyphid *Plesioglyphus* [2], it has never been formally described. *Oriboglyphus maorianus* is particularly interesting due to its retention of several ancestral traits, including three pairs of genital papillae, five pairs of genital setae, a distinct mouth and pharynx, and rudimentary chelicerae. Here, we provide a detailed description of *O. maorianus,* focusing on these traits, and clarify its ecological characteristics. These data may help determine whether schizoglyphid phoretic nymphs should be classified as deutonymphs, akin to those of other Astigmata, or as tritonymphs, supporting the early-burst hypothesis of life cycle diversification in Astigmata. The aim of this study is to provide a comprehensive description and comparative analysis of *O. maorianus* from *Stolotermes ruficeps* in New Zealand, highlighting the relevance of this new evidence for addressing evolutionary hypotheses for the early evolution to phoresy-based metamorphosis in Astigmata. These hypotheses, however, will be tested in a separate publication using quantitative modeling approaches, as the present study focuses on descriptive and comparative morphological analyses that provide the necessary foundation for such work.

## 2. Materials and Methods

Five specimens were found in galleries of the New Zealand wetwood termite *Stolotermes ruficeps* (family Stolotermitidae), in a *Pinus radiata* log in Thames, New Zealand (Dickson Holiday Park, Tinker Trail, 37°06′42.7″ S 175°31′22.1″ E). The mite specimens were ethanol-washed in a Petri dish from alate females, workers, and immature termites, and were subsequently collected from the bottom of the dish.

Images were captured from multiple focal planes and assembled using Helicon Focus Pro 7.6.4 (algorithm B, occasionally A), with subsequent manual editing (retouching) of any misaligned regions. Partially overlapping images were merged into a full panorama in Adobe Photoshop 22.2.0. Line drawings were created in Photoshop 22.2.0 using microphotographs as the background. The background images were taken using a Axiocam 506 color digital camera (Carl Zeiss, Oberkochen, Germany) attached to a Axio Imager A2 compound microscope (Carl Zeiss, Oberkochen, Germany) equipped with bright field and differential interference contrast (DIC) optics.

Morphological terminology. Idiosoma chaetotaxy follows Griffiths et al. [30]; the terminology of coxisternal setae follows Norton [31]; gnathosomal morphology follows Grandjean [32]; for appendages, the chaetotaxy and solenidiotaxy follow Grandjean for palps [33] and legs [34]. Comparisons with *Schizoglyphus biroi* are based on new illustrations of a paratype specimen in OConnor (2009) [17].

Two paratype specimens were used for semi-destructive DNA extraction and sequenced using Illumina (USA) 2 × 150 bp technology. Our taxonomic action and classification of the new genus and species as a member of Schizoglyphidae are based, among other things, on molecular genetics, details of which to be published elsewhere, focused on molecular and phylogenetic analyses.

## 3. Results

### 3.1. Taxonomy

Family Schizoglyphidae Mahunka, 1978

Genus *Oriboglyphus* Klimov and Kolesnikov, gen. n.

urn:lsid:zoobank.org:act:37BC59EC-270E-491D-B2FC-39C2AC465BB3

Diagnostic description. *Phoretic tritonymph*. Gnathosoma well developed, with subquadrate subcapitular remnant and 3-segmented palpi, setae *a* and *or*_2_ present, filiform; setae *h* and *or*_1_, present, alveolar. Labrum present, short. Anteriodorsal wall of gnathosoma bilobed. Supracoxal setae of palps present, wide. Long pharynx present. Body rounded, ratio of hysterosoma/propodosoma 1.23. Anterior part of propodosomal shield narrowed, extended forward, forming rostrum. Dorsal idiosoma reticulate. Setae *vi* and *ve* present. Dorsal setae heteromorphic—long extended and short filiform, setae *c*_3_ absent. Coxal fields I, II and III closed; coxal fields IV open. Five pairs of genital setae and three pairs of genital papillae. Anus situated between *ad*_3_ and *ad*_1+2_. Tarsal setae *aa* I-II, *ba* I, *wa* I-II, *v* IV present, setae *ba* II, *ra* I-II, *u* I-IV, *v* I-III, *nG* III, *wF* IV absent, setae *p* I-IV and *q* I-II vestigial. Solenidia ω_1_ I-II, ω_2_ I, ω_3_ I-II present, solenidion σ I single. Famulus situated in cuticular depression.

Type species: Oriboglyphus maorianus sp. n.

Remarks. The new genus differs from *Schizoglyphus*, the only known recent genus in the family Schizoglyphidae as follows: the body is rounded (elongated in *Schizoglyphus*), dorsal idiosomal setae differ in shape, band-shaped and pectinated versus filiform and smooth (all setae smooth and filiform in *Schizoglyphus*), five pairs of genital setae (four pairs in *Schizoglyphus*), the anus is situated between *ad_3_* and *ad_1+2_* (between *ad_1+2_* in *Schizoglyphus*), coxal fields I-II closed (open in *Schizoglyphus*) and a reticulated dorsum (punctate in *Schizoglyphus*). The new genus differs from fossil *Plesioglyphus* as follows: subcapitular remnant small, distinctly not reaching femora I (larger, reaching femora I in *Plesioglyphus*), coxal fields I closed (open in *Plesioglyphus*).

As noted in Sendi et al. (2025) [2], the presence of chelicerae remnants in phoretic stages has been confirmed only in two genera of astigmatid mites: *Oriboglyphus* gen. n. and *Levantoglyphus* Klimov et al., 2021 (family Levantoglyphidae). Specifically, *Oriboglyphus* exhibits cheliceral seta *cha* and sclerotized bodies interpreted as chelicerae with distinct internal tendons, while *Levantoglyphus* has rudimentary chelicerae enabling food shredding. Both genera also share additional gnathosomal similarities, including three-segmented palps, the presence of *ul’* and *ul”* setae on palpal tarsi, setae *a* and *ep* on the subcapitular remnant, and a podocephalic canal, as well as the presence of seta *aa* II. These similarities suggest a phylogenetic affinity between Levantoglyphidae and Schizoglyphidae. However, a key distinction between the families remains the more developed gnathosoma in *Levantoglyphidae* (due to the presence of well-formed chelicerae) and the single pair of genital setae in *Levantoglyphus*. Nevertheless, limitations in observing the morphology of fossil specimens leave the number of genital papillae uncertain, complicating the confident interpretation of *Levantoglyphus sidorchukae* as an exclusive phoretic deutonymph [9] rather than a phoretic tritonymph, analogous to *Oriboglyphus* gen. n.

Etymology. *Oriboglyphus* is a contraction of Oribatida (soil mites) and glyphus (from Greek γλύφω, to carve, to engrave), a common stem used for name formation in Astigmata. The name reflects the blend of traits from both Oribatida and Astigmata in this new genus. Gender masculine.

Oriboglyphus maorianus sp. n.

urn:lsid:zoobank.org:act:4D84E044-F89A-407F-8BD2-58A2EE6D6D7D

Material. Holotype (phoretic tritonymph)—New Zealand: Thames, Dickson Holiday Park, beginning of Tinker trail, *Pinus radiata* log, galleries of *Stolotermes ruficeps* (Blattodea: Kalotermitidae), ethanol wash of all termite stages, including alate adults, 37°06′42.7″ S 175°31′22.1″ E, 19 December 2022, P. Klimov, PBK 22-1201-037. The holotype is deposited at the New Zealand Arthropod Collection, Landcare Research, Auckland, New Zealand (NZAC).

Paratypes: 2 phoretic tritonymphs (DNA extraction vouchers)—same data, deposited at the United States National Museum of Natural History, Smithsonian Institution, Washington, DC, USA (USNM); 1 phoretic tritonymph (preserved in ethanol, not mounted)—same data, deposited at the Universidade Federal de Minas Gerais, Pampulha, Belo Horizonte, MG, Brasil (UFMG), 1 phoretic tritonymph (preserved in ethanol, not mounted)—same data, deposited at the Zoological Institute, Russian Academy of Sciences, Saint Petersburg, Russia (ZISP); 1 phoretic tritonymph—New Zealand: Auckland, Kaipara Flats, alate Stolotermes ruficeps, Sept 2007, S.J. Bennett, IDC Ref. 09/2007/1016. Deposited at the Plant Health & Environment Laboratory, Ministry for Primary Industries, Auckland, New Zealand (PHEL), 3 phoretic tritonymphs—same data, 15 Mar 2007; IDCf Ref 09/2007/802. Deposited: PHEL (2 slides), UMMZ (1 slide).

Additional material (not mounted): Phoretic tritonymphs (not counted)—New Zealand: Auckland, Kaipara Flats, alate Stolotermes ruficeps, 05 Apr 2007, S.J. Bennett. Deposited: PHEL, phoretic tritonymphs (not counted)—New Zealand, Auckland, Kaipara Flats, 34 Old Woodcocks Rd, 26 Feb 2008, C. Inglis; IDC Ref. 09/2008/960. Deposited: PHEL.

**Figure 1:**
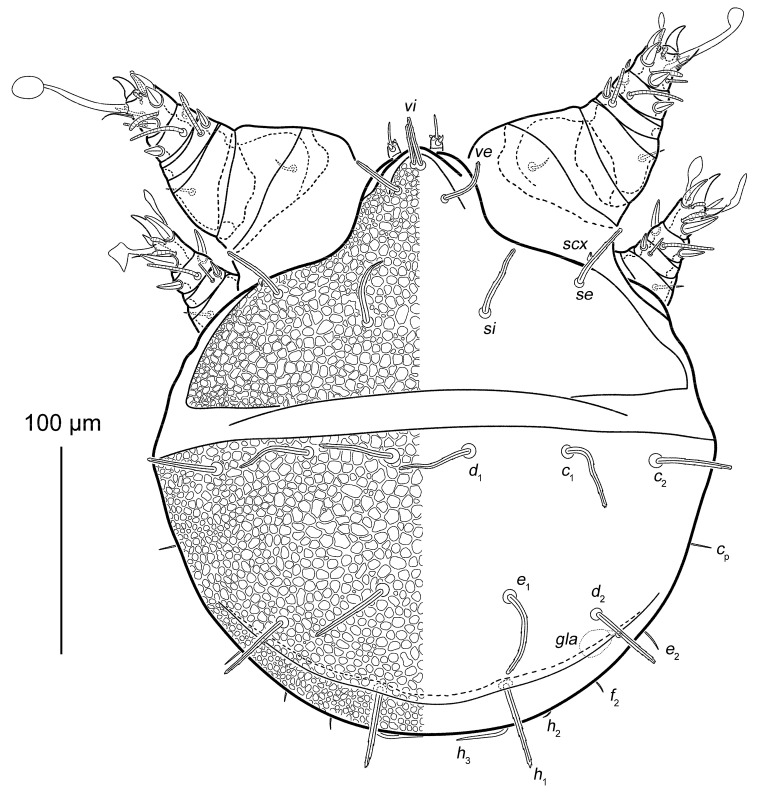
*Oriboglyphus maorianus* gen. et sp. n., dorsal view.

**Figure 2:**
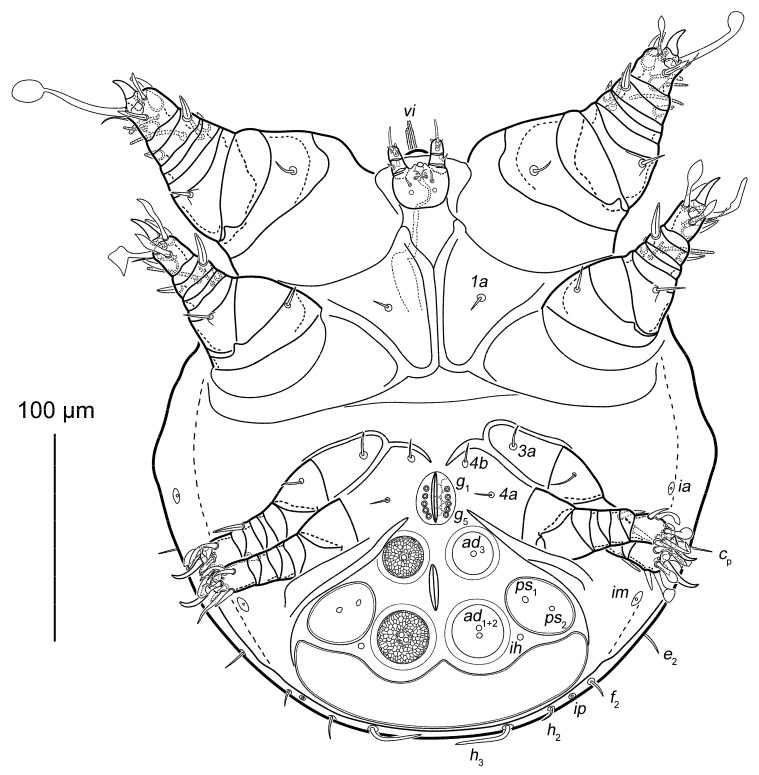
*Oriboglyphus maorianus* gen. et sp. n., ventral view.

**Figure 3:**
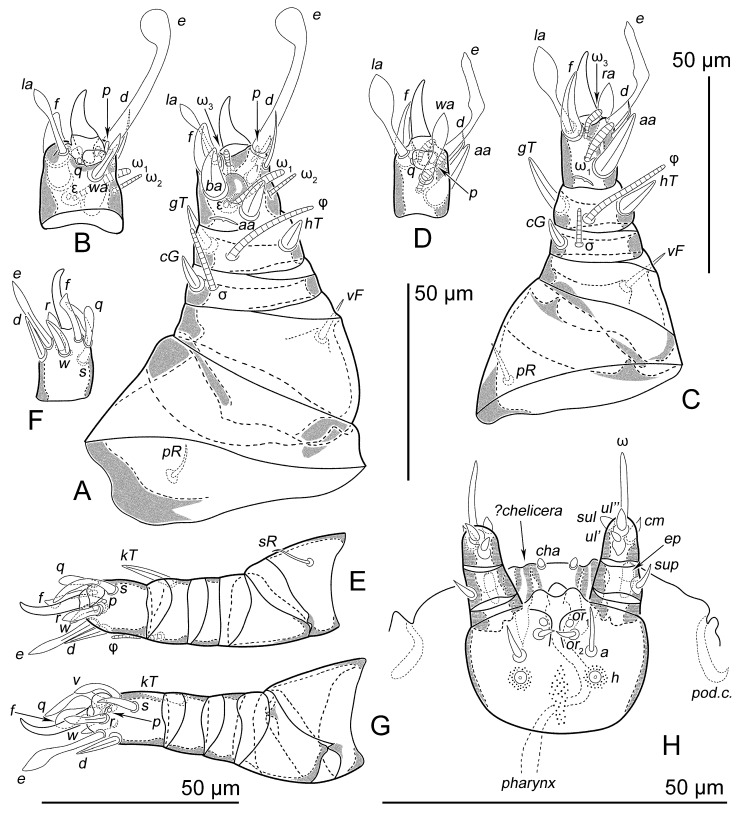
*Oriboglyphus maorianus* gen. et sp. n.: (**A**)—leg I, dorsal view; (**B**)—tarsus I, ventral view; (**C**)—leg II, dorsal view; (**D**)—tarsus II, ventral view; (**E**)—leg III, ventral view; (**F**)—tarsus III, dorsal view; (**G**)—leg IV, ventral view; (**H**)—gnathosoma, ventral view.

**Figure 4:**
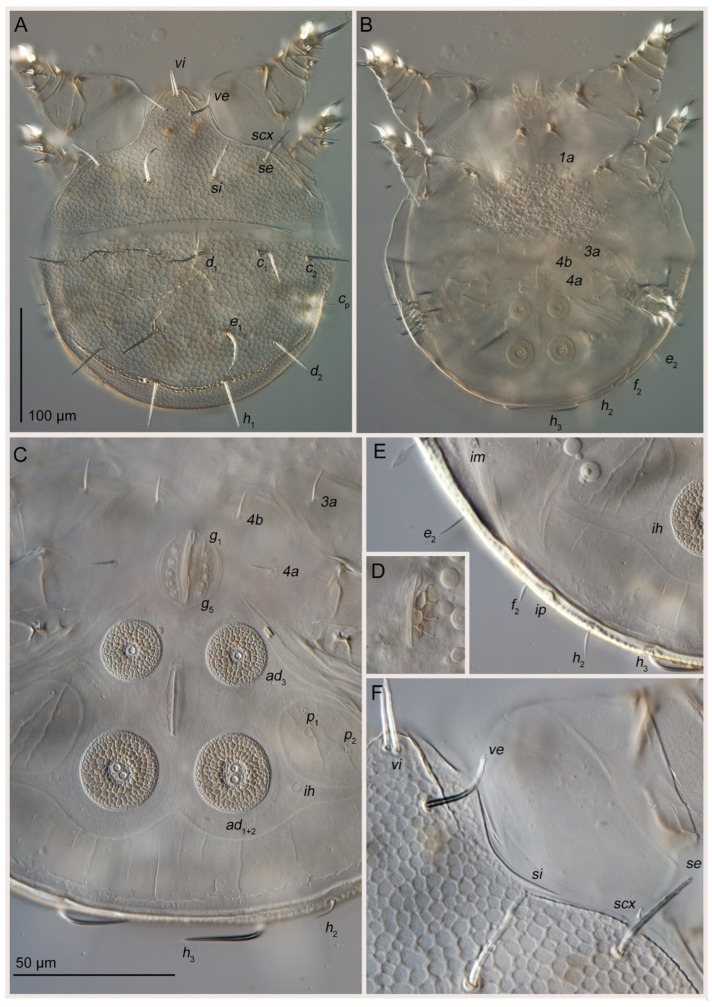
*Oriboglyphus maorianus* gen. et sp. n., DIC images: (**A**)—dorsal view; (**B**)—ventral view; (**C**)—anogenital region; (**D**)—genital papillae; (**E**)—posterior-lateral region, ventral view; (**F**)—prodoral shield, part, dorsal view.

**Figure 5:**
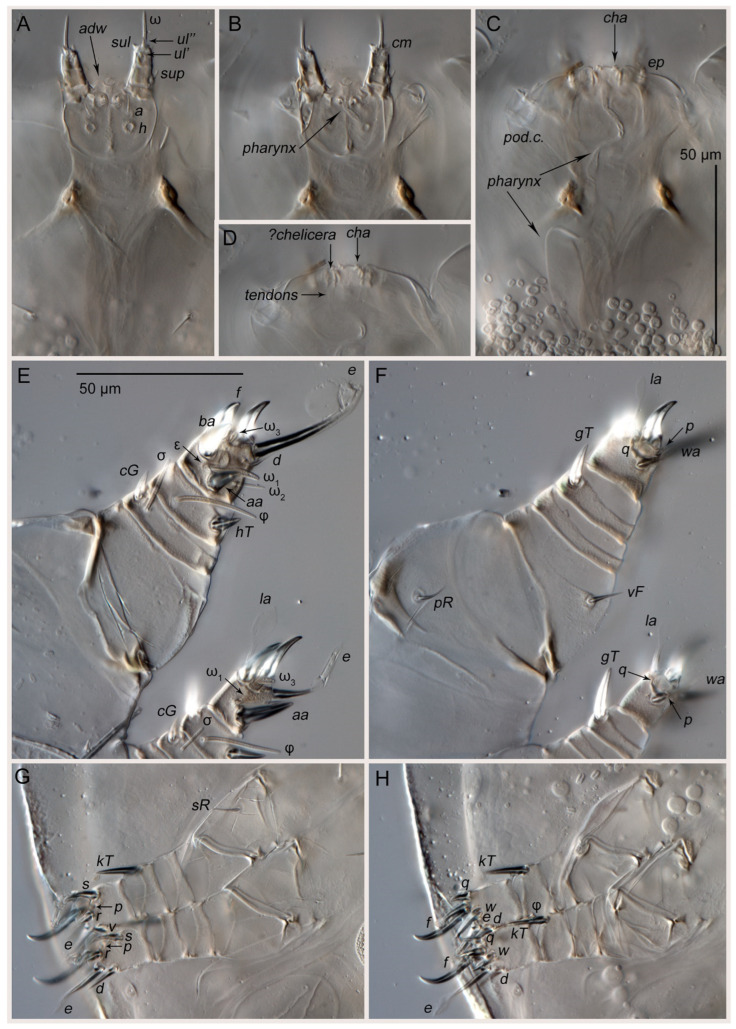
*Oriboglyphus maorianus* gen. et sp. n., DIC images: (**A**–**D**)—gnathosoma and anterior part of propodosoma, at different focal planes; (**E**)—leg I and tarsus II, dorsal view; (**F**)—leg I and tarsus II, ventral view; (**G**)—legs III–IV, ventral view; (**H**)—legs III–IV, dorsal view.

Phoretic nymph (Figure 1, Figure 2, Figure 3, Figure 4 and Figure 5). Gnathosoma (Figure 3H and Figure 5A–D) well developed, with subquadrate subcapitular remnant (22 × 27) and 3-segmented palpi (17). Subcapitular remnant with a ventromedial sclerite, one pair of weakly widened setae *a* (6) and one pair of rounded alveoli of *h*, situated posterior to setae *a*. Genae with two rounded lobes bearing two adoral setae (*or*): *or*_1_ alveolar; *or*_2_ (2), filiform, widened. Bases of *or*_1_ appear to continue dorsally resembling superior commissure of mouth. Anteriodorsal wall (*adw*) of gnathosoma bilobed. This bilobed wall can be interpreted as dorsal portion of lateral lips, while adoral lobes can be interpreted as adoral sclerite *sensu* Hammen ([35], Figure 91) [10]. Rutellum absent. Labrum short, rounded, situated near small oral opening, leading into pharynx. Pharynx long and narrow, reaching coxal setae *1a*, significantly expanded in this area. Palpal setation 0–1–4 + ω. All palpal setae spiniform, but *sup* (4) slightly longer and narrower than others. Palptarsal setae *ulʹ* (2) and *ulʺ* (3) ventral, *sul* (2) paraxial, *cm* (4) dorsal. Palptarsal solenidion ω slightly shorter than palps, straight. Supracoxal setae of palps (*ep*) present, short (4), wide, with serrated blunt apex. Apices of palps projecting beyond anterior edge of propodosoma. Podocephalic canal (*pod.c.*), present, situated on each side of gnathosoma. Above subcapitulum, there is a pair of very short (1) apical, spiniform, cheliceral setae (*cha*); on each side of *cha* there are sclerotized bodies (presumably cheliceral homologues), with distinct internal tendons (Figure 5D). Most of subcapitular remnant and palps finely punctate; medial area and rounded area around alveolae *h* coarsely punctate.

Body rounded (Figure 1, Figure 2 and Figure 4A,B), 290 × 270, length/width ratio 1.07. Propodosoma 130, hysterosoma 160, ratio of hysterosoma/propodosoma 1.23 (n = 1). Dorsal idiosoma with reticulate pattern, consisting of rounded cells; space inside cells finely punctuate (Figure 1 and Figure 4A,F). Anterior part of propodosomal shield with a large rostrum (Figure 4A). Rostrum with paired vertical setae (*vi* and *ve*); setae *vi* (22–26) adjacent, bases in common area of unsclerotized cuticle; setae *ve* (28) distinctly posterior and lateral to *vi*. Ocelli absent. Sejugal furrow distinct. Internal scapular setae *si* situated medially, slightly posterior than external scapular setae *se*; *se* lateral. Hysterosomal shield with 11 setae: *c*_1_ (40), *c*_2_ (40), *c*_p_ (12), *d*_1_ (38), *d*_2_ (40), *e*_1_ (44), *e*_2_ (12), *f*_2_ (10), *h*_1_ (47), *h*_2_ (12) and *h*_3_ (30). Setae *d*_1_ situated on same transverse level with *c*_1_. Dorsal setae *vi*, *ve*, *si*, *se*, *c*_1_, *c*_2_, *d*_1_, *d*_2_, *e*_1_ and *h*_1_ long, flattened, with median ridge; finely pectinate; setae *c*_p_, *e*_2_, *f*_2_ and *h*_2_ short, thin, smooth, filiform; setae *h*_3_ wide, lanceolate, smooth, without median ridge, curved at base. Setae *scx* (5) short, with bifurcated tip. Opisthonotal gland openings (*gla*) situated between bases of *d*_2_ and *e*_2_. Cupules *ia*, *im*, *ip* and *ih* present; cupules *ip* dorsal, situated between bases of *f*_2_ and *h*_2_; cupules *ia* ventral, anterior to transverse level of trochanters IV; cupules *im* ventral, posterior to transverse level of trochanters IV; cupules *ih* ventral, lateral to suckers *ad*_1+2_ of attachment organ. All cupules (except *ih*) are surrounded by a slightly sclerotized ring.

Surface of coxal fields smooth, without any well-developed pattern (Figure 2 and Figure 4B,C). Coxal fileds I-III closed, coxal fields IV open. Anterior apodemes of coxal fields I fused, forming a sternum. Posterior end of sternum bifurcated, fused with ends of anterior apodemes II. Distance between end of sternum and ventrogenial shield much shorter than length of sternum. Posterior apodemes II thin, weakly-developed, merged with anterior apodemes II. Apodemes II not fused with apodemes III. Coxal fields III not fused with each other, with a short gap between them, fused to anterior apodemes IV. Anterior apodemes IV not fused with each other, with a short gap between them. Ventrum absent. Coxal setae *1a*, *3a*, *4a* and *4b* filiform, short (8–11); *1a*, *4a* and *4b* smooth, *3a* finely pectinate. Setae *c*_3_ absent. Genital opening (Figure 4C) situated between trochanters III-IV, with five flattened, button-shaped genital setae (*g*_1–5_). Three pairs of rounded genital papillae (Figure 4D). Attachment organ (Figure 4C,E) shaped as large rounded-corner triangle, with longest side situated near posterior body margin, 115 long, 160 wide (length/width ratio 0.7). Suckers *ad*_3_ and *ad*_1+2_ round, large, with sclerotized reticulate “crocodile-skin” pattern; alveoli well-developed. Centers of suckers *ad*_3_ and *ad*_1+2_ arranged in a rectangle; anus positioned at center. Setae *ps*_1_ and *ps*_2_ form a common cuticular sucker, with distinct alveoli of *ps*_1_ and *ps*_2_; sucker lateral to *ad*_1+2_. Setae *ps*_3_ absent. Elongated unpaired transverse cuticular sucker situated on posterior edge of attachment organ (Figure 4C,E).

All legs (Figure 3A–G and Figure 5E–H) shorter than half the body length. Legs I (150) distinctly larger and wider than legs II (95) and legs III-IV (80). Leg I conical, leg II nearly conical. Leg setation (Table 1): trochanters 1–1–1–0, femora 1–1–0–0, genua 1 (1)–1 (1)–0–0, tibiae 2 (1)–2 (1)–1 (1)–1, tarsi 9 (3 + ε)–8 (2)–8–9. Trochanters I–III each with a filiform seta (*pR* I, *pR* II, *sR* III, respectively). Femoral setae *vF* I, II weakly spiniform. Genual setae *cG* I and II spiniform; setae *mG* I-II and *nG* III absent. Tibial setae *gT* I-II and *hT* I-II spiniform, *gT* I-II longer than *hT* I-II. All pretarsi with hooked empodial claws arising near tarsal apices and connected to short, paired condylophores. Tarsus I with three spiniform setae (*aa*, *ba*, *f*), one filiform seta *d*, two lanceolate setae (*wa* and *la*), one modified setae *e* (wide leaf-shaped tips), two small vestigial setae (*p* and *q*), and three solenidia (ω_1_, ω_2_, ω_3_). Tarsus II with two spiniform setae (*aa* and *f*), one filiform seta *d*, two lanceolate setae (*wa* and *la*), one modified setae *e* (wide leaf-shaped tips), two small vestigial setae (*p* and *q*), and two solenidia (ω_1_ and ω_3_). Solenidia ω_1_ and ω_3_ I-II with rounded apices. Solenidion ω_1_ I situated slightly anterior to *aa* in a small depression; ω_1_ II situated slightly anterior to *ba* in a small depression. Solenidia ω_3_ I-II shorter than ω_1_, situated near *f*. Solenidion ω_2_ I bacilliform, antiaxial. Famulus ε small, spiniform, situated in cuticular depression next to ω_1_. Lyrifissures present on basal part of tarsi I and II. Tarsus III with two spiniform, sword-shaped setae (*d* and *w*), five slightly lanceolate setae (*e*, *f*, *r*, *s*, *q*) and one small vestigial seta *p*. Tarsus IV similar to tarsus III, but it has setae *v*, which is slightly lanceolate. Solenidia φ I-III slightly tapering, shorter than total length tibia-tarsus, φ IV absent. Solenidia σ I and II bacilliform, distinctly shorter than φ; σ III absent.

Etymology. The species epithet *maorianus* is a Latinized adjective (masculine gender), meaning “of the Māori,” and is intended to highlight this interesting species as indigenous to Aotearoa (New Zealand).

**Plesiomorphic and apomorphic traits of schizoglyphids**. Schizoglyphids exhibit a number of plesiomorphic traits that are shared with their oribatid ancestors, Malaconothridae (Desmonomata), based on morphology [31], or as the sister group to the Desmonomata + Brachypylina clade, with Mixonomata as the outgroup, according to molecular evidence [36,37]. For instance, *Schizoglyphus* and *Oriboglyphus* have several structures that suggest close similarities to one of these two oribatid lineages: three-segmented palps (two-segmented in the remaining Astigmata, 2–5 segments in other Oribatida), the palptarsus with setae *sul* (absent in the remaining Astigmata, present in other Oribatida), the gnathosoma has two pairs of adoral setae *or_1_*-*or*_2_ (absent in the remaining Astigmata, present in Oribatida) and setae *h* (absent in the remaining Astigmata, present in Oribatida); four (*Schizoglyphus*) or five (*Oriboglyphus*) pairs of genital setae (0–1 pairs in the remaining Astigmata, 1–24 in Oribatida, but Mixonomata and Desmonomata commonly have 7–9 (4 of deutonymph and 6–7 of tritonymph) and 8–14 (3–7 in deutonymph and 5–10 of tritonymph) pairs, three pairs of genital papillae (two pairs in the remaining Astigmata, three pairs in most Oribatida), and two solenidia on tarsus II, ω_1_ and ω_3_ (many of the remaining Astigmata have ω_1_ only, Oribatida have two solenidia, ω_1_ and ω_2_, so this character state can also be interpreted as an apomorphy).

Apomorphic traits of Schizoglyphidae include setae *ps_1_* and *ps_2_* represented by alveoli and situated on enlarged anterior cuticular suckers. In all other Astigmata, setae *ps_1_* and *ps_2_*, are each modified into a separate conoid. Schizoglyphids also have a single, very broad posterior sucker curving around posterior half of attachment organ; in all other Astigmata, posterior cuticular suckers are either absent or represented by one paired and two unpaired suckers [30,38]. Proral tarsal setae *q* I-II and *p* I-IV vestigial in *Oriboglyphus* and *Schizoglyphus* (usually well developed in other Astigmata).

This suite of plesiomorphic traits, along with the above apomorphic character states, suggests that this family is the sister group to remaining Astigmata.

#### Key to Genera and Species of Schizoglyphidae *

(phoretic nymphs, adult stages unknown)

1. Subcapitular remnant larger, reaching femora I. Phoretic on termite *Lebanotermes veltzae*. Lebanese amber (Early Cretaceous) …† *Plesioglyphus lebanotermi* Sendi, Klimov, Kolesnikov, Káčerová, Bonino, Azar et Robin, 2025

- Subcapitular remnant smaller, not reaching femora I … 2

2. Body elongated, with punctate dorsum. Coxal fields I-II open. Dorsal idiosomal setae uniformly smooth and filiform. Four pairs of genital setae. Anus situated between *ad_1_*_+2_. Originally described from the tenebrionid beetle *Dioedus tibialis*, though this association is likely accidental, with actual associations involving termites. Western New Guinea … *Schizoglyphus biroi* Mahunka, 1978

- Body rounded, with reticulated dorsum. Coxal fields I-II closed. Dorsal idiosomal setae differ in shape: band-shaped and pectinated versus filiform and smooth. Five pairs of genital setae. Anus situated between *ad_3_* and *ad_1+2_*. From nest of termite *Stolotermes ruficeps*. New Zealand … *Oriboglyphus maorianus* gen. and sp. n.

* all genera are monotypic.

### 3.2. Phoretic Associations of Oribatida

**Figure 6:**
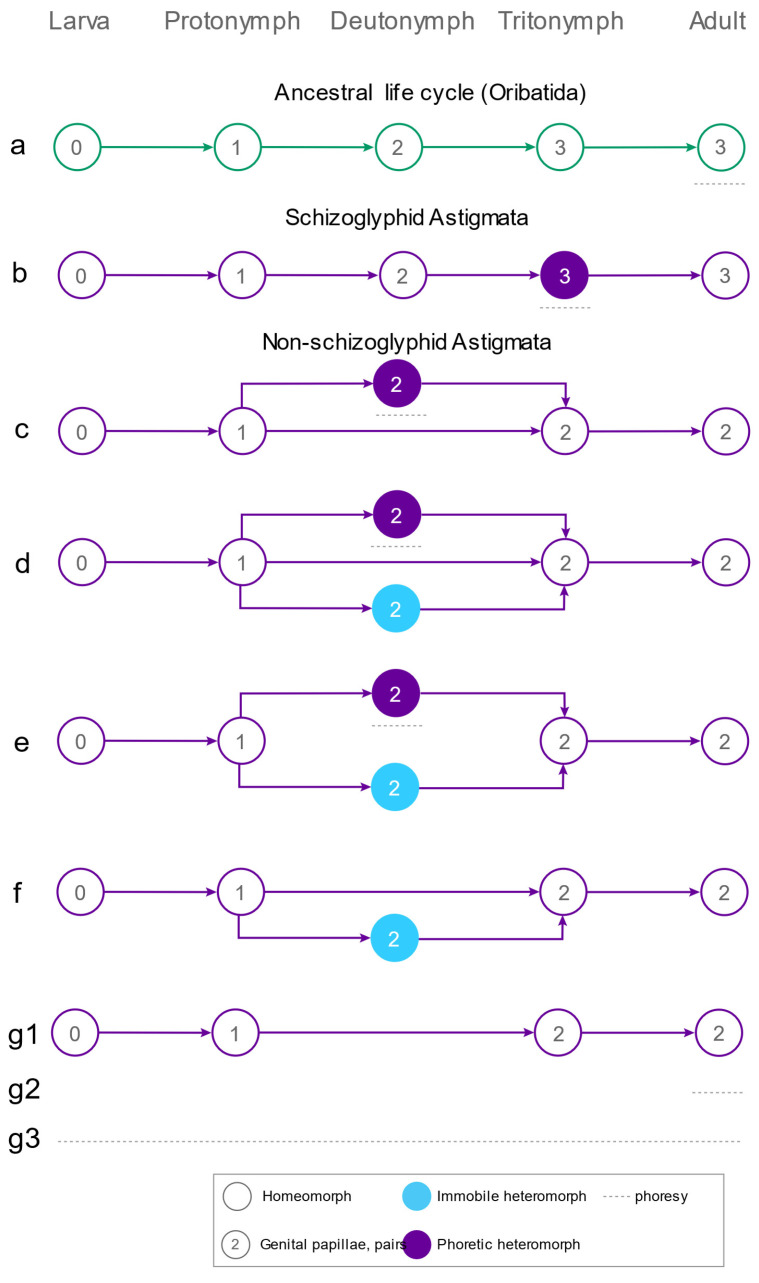
Life cycles of Oribatida and Astigmata. (**a**)—ancestral trinymphal life cycle (Oribatida) lacking a specialized nymphal phoretic stage but unspecialized adults can be phoretic; (**b**)—putative life cycle of Schizoglyphidae (Astigmata) with a specialized tritonymphal phoretic stage; (**c**–**f**)—life cycles of non-schizoglyphid Astigmata with a specialized facultative deutonymphal phoretic/survival stage; (**g**)—life cycle of non-schizoglyphid Astigmata lacking a deutonymphal stage.

Although oribatid mites are generally considered non-phoretic, this is partly because the phoresy is mostly behavioral and hence cryptic. Numerous instances phoresy have been documented, primarily involving adults (males and females or only females) with no or minimal morphological specialization for phoresy, and always apparently retaining their typical trinymphal life cycle (Figure 6a). Most phoretic associations involve the wood-associated beetle families Passalidae, Scarabaeidae, and the curculionid subfamily Scolytinae. Of them, the most numerous finds of phoretic oribatid mites were discovered on beetles of the family Passalidae—at least 18 species of mites (some identified only to the genus level) are known to be associated with 26 passalid species [39,40,41,42,43,44]. Commonly found genera include *Mesoplophora* (Mesoplophoridae), *Paraleius* (Hemileiidae), *Protoribates* (Protoribatidae), *Malaconothrus* (Malaconothridae), *Oppia* (Oppiidae), and *Scheloribates* (Scheloribatidae). The bark beetle *Dendroctonus frontalis* Zimmermann, 1868 (Scolytinae) hosts several oribatid species, including *Paraleius leontonycha (Berlese, 1910)*, *Dometorina* sp., and *Tectocepheus velatus sarekensis Trägårdh, 1910* [39]. Other scolytine beetles such as *Dryocoetes affaber LeConte, 1876*, *Hylastes nigrinus LeConte, 1868*, *Hylastes salebrosus Eichhoff, 1868* and *Pityokteines* spp. also carry phoretic *Paraleius* spp. and *Phauloppia lucorum* (Koch, 1841) [39,45,46,47]. Phoresy of *Paraleius leontonycha* has been recorded on *Ips typographus* Linnaeus, 1758 [48]. Oribatids have also been documented on longhorn beetles (*Tragosoma* sp.; Cerambycidae), including *Scheloribates* spp. [39]; *Euscheloribates samsinaki Kunst, 1958* (Scheloribatidae) associated with the scarab beetle *Osmoderma eremita Scopoli, 1763* [49]; *Eremella (Licnocepheus) reticulatus* (Woolley, 1969) (Eremellidae) has been recorded on *Lacon discoideus* (Weber, 1801) and *Lacon marmoratus* (Fabricius, 1801) (Elateridae), with both male and female mites participating in phoresy [50]; *Eremella ryabinini* Ermilov et Abramov, 2023 has been recorded on *Amphotis marginata* (Fabricius, 1781) (Nitidulidae) [51]. *Paraleius sp. has also been found phoretic on Pantophthalmus heydeni Wiedemann, 1830 (Diptera), and Mesoplophora sp. on Salganea passaloides (Walker, 1868) (Dictyoptera)* [39].

In addition to insect associations, oribatid mites occasionally use vertebrates as carriers. *Benoibates* (Oripodidae) were recorded on the frog *Pristimantis ornatissimus* (Despax, 1911) (Strabomantidae), representing the only known amphibian association. More significantly, *Benoibates* nymphs are the life stages which have become behaviorally specialized for phoresy. This parallels the Astigmata and provides one of the very few examples involving immature stages [52]. Oribatids have also been found in the plumage of birds [53,54,55], although their role is unclear and may involve facultative phoresy or passive/opportunistic transport. In mammals, a few cases of oribatid mites clinging to fur have been documented [56], possibly as accidental occurrences or rare examples of dispersal behavior. In striking contrast to many of the Astigmata, no known oribatid mite seems to have developed an obligatory relationship with vertebrates or their nests [39].

These findings collectively suggest that phoresy among Oribatida is taxonomically widespread, based on the trinymphal life cycle (Figure 6a), predominantly involves the adult stage, and is mostly linked to wood-associated insect hosts—with occasional dispersal on vertebrates, including birds, mammals, and amphibians. At the same time, those species of oribatid mites that exhibit phoretic behavior have developed little in the way of morphological specializations for attachment to the host [39]. This is in dramatic contrast to mites belonging to Prostigmata and Astigmata, many of which show a variety of diverse adaptations for attachment to hairs and integument of insects and vertebrates [17,57,58,59,60,61].

### 3.3. Diversity of Phoresy-Related Life Cycles of Astigmata

Unlike Oribatida, non-schizoglyphid Astigmata exhibit an exceptional diversity of life cycle strategies (Figure 6c–g). These mites not only modulate the production of dispersal stages in response to environmental conditions, but also exhibit developmental plasticity in metamorphic outcomes. Depending on ecological context, development may be channeled toward dispersal, survival, or reproduction and resource utilization [17] (Figure 6c–g).

The most common life cycle involves a binary developmental switch, in which the protonymph can molt either directly into the tritonymph or into a phoretic deutonymph, which later molts into the tritonymph (Figure 6c). This common life cycle pattern is exemplified by various members of Astigmata, such as the Acaridae (e.g., *Naiadacarus*, *Sancassania*, *Rhizoglyphus*), Histiostomatidae (e.g., *Histiostoma*, *Hormosianoetus*, *Nepenthacarus*), Chaetodactylidae except *Chaetodactylus* (e.g., *Sennertia*, *Roubikia*), Winterschmidtiidae, Lemanniellidae, and Hypoderatidae [38,62,63,64,65,66,67,68,69,70,71,72].

A more complex but common version of this pattern involves a ternary (i.e., three-way) ontogenetic switch, in which the protonymph has three potential developmental trajectories: (1) direct molt to the tritonymph, (2) molt to a phoretic deutonymph, or (3) molt to a non-phoretic, often immobile (heteromorphic) deutonymph. The non-phoretic deutonymph functions as a survival stage under adverse conditions—for instance, in *Chaetodactylus* ssp, it may remain dormant within a bee nest cavity, awaiting its reuse by a subsequent host (Figure 6d). The ternary ontogenetic switch life cycle has been documented in species of the genus *Chaetodactylus* (Chaetodactylidae), *Glycyphagus privatus* Oudemans, 1903, *G. ornatus* Kramer, 1881, *Baloghella melis* Mahunka, 1963 (Glycyphagidae), *Alabidopus asiaticus* Lukoschus et al. 2009 (Chortoglyphidae), and presumably *Hericia* sp. (Algophagidae) [38,73,74,75,76,77,78].

The ternary switch life cycle can itself be modified in various ways. For instance, in *Tensiostoma veliaphilum* Wurs et Kovac, 2003 (Histiostomatidae), only the two deutonymphal loops are retained—phoretic and immobile—with the direct molt from protonymph to tritonymph absent (Figure 6e), so either type of deutonymph is obligatory. This mite inhabits the water-air interface in bamboo internodes filled with water and uses the Oriental semiaquatic bug *Lathriovelia* (Veliidae) as its phoretic host. Interestingly, the immobile deutonymph in *T. veliaphilum* does not appear to be associated with adverse conditions [79].

In another modification of the ternary switch, the phoretic deutonymph is lost, and only the immobile survival deutonymph remains (Figure 6f). This pattern is found in *Acarus immobilis* (Acaridae) and *Glycyphagus* (*Lepidoglyphus*) *destructor* (Schrank, 1781) [80,81,82].

At the other end of the spectrum is the simplest life cycle, in which the protonymph always molts directly into the tritonymph, with no specialized dispersal or survival deutonymphal stages (Figure 6(g1)). This life cycle is typical of species inhabiting predictable, continuous environments such as littoral habitats (Hyadesiidae) [83,84]. Another ecological group that retains this simple life cycle includes mites inhabiting discontinuous ephemeral environments but which predominantly rely on dispersal as homeomorphic stages either through passive aerial transport or through active locomotion. An example is *Tyrophagus putrescentiae* (Schrank, 1781) (Acaridae), a fast-moving species with long idiosomal setae capable of dispersing without a specialized deutonymphal stage (Figure 6(g1)) [85,86].

A similar simplification of the binary switch life cycle is found among mites that are permanently associated with vertebrate or invertebrate hosts. In such cases, the dispersal deutonymph is often absent, as the host provides both a habitat and means of dispersal. These mites are transmitted vertically or through direct contact between hosts. Examples include many Psoroptidia associated with birds and mammals [67,87,88,89], *Ewingia* and similar genera (on decapod crustaceans, such as hermit and fresh water crabs) [90,91], most Canestriniidae except *Megacanestrinia* (beetles), Heterocoptidae, and *Sennertia vaga Klimov and OConnor, 2008* (Chaetodactylidae), which disperses using its typical developmental stages on large carpenter bees (*Xylocopa*), while reproduction likely occurs both on the host and within the nest [38] (Figure 6(g1)). Some such lineages, such as (Hemisarcoptidae) and *Rosensteinia* (Rosensteiniidae), have become truly parasitic [92]. In a distinct case, some bird skin parasites such as those in the Epidermoptidae have also lost the deutonymphal stage, but disperse via females that are morphologically adapted for transport. These females attach to hippoboscid flies or lice and behave parasitically during transport [93,94] (Figure 6(g2)), blurring the line between phoresy (typically harmless) and parasitism (which involves host damage).

*Finally, a third ecological group comprises mites that disperse both* via *hosts and on their own* despite having lost the phoretic deutonymph. *Pyroglyphidae, for example,* presents an interesting case in which mites must still disperse among vertebrate nests—their natural habitat—but have irreversibly lost the phoretic deutonymphal stage, a trait eliminated in their parasitic ancestors within Psoroptidia [95]. Instead, they disperse on the plumage, hair, or skin of nest-building animals, while short-range dispersal (such as adjacent nests in communal or social animals) is accomplished by walking or areal currents (Figure 6(g1)). Another example is *Tyrophagus putrescentiae*, which primarily disperses on its own (see above), but occasionally can be found (behaviorally) phoretic on insects or vertebrates (Figure 6(g3)).

## 4. Discussion

In Astigmata, life cycle modifications have evolved according to the strong benefits that accrue from dispersal, resulting in the development of a specialized immature phoretic stage [92]. As noted in the Introduction, phoresy occurring earlier in the life cycle allows mites to leave depleted resources more quickly, thereby increasing their chances of locating suitable new habitats and successfully establishing new populations. In contrast, phoresy at later ontogenetic stages—closer to sexual maturity—may enable more immediate reproduction upon arrival, offering a competitive advantage by accelerating population growth during the initial and often most critical phase of colonization. Given that Astigmata have one prelarval, one larval, three nymphal stages, and one adult stage, multiple developmental pathways for phoresy are theoretically possible. Any of these stages could, in principle, adopt a phoretic role, offering ontogenetic and ecological flexibility and enabling the diversification of dispersal strategies. For example, adult phoresy is seen in their oribatid ancestors [39], in which some adult mites use a locking mechanism to attach to hairs of their phoretic host. The locking mechanism typically is part of a defensive strategy: the mite curls into a compact form, with sclerotized shields protecting the exterior and vulnerable structures like the legs retracted and safeguarded internally; the locking mechanism serves to maintain this protective posture [96,97]. In Astigmata, adult phoresy is known in a few derived lineages but it is not based on locking mechanisms [38].

The recent discovery of *Oriboglyphus maorianus*, an extant schizoglyphid mite [2], presents a rare opportunity to explore the early diversification of phoresy-related metamorphosis in Astigmata. Morphologically, *O. maorianus* retains several plesiomorphic traits—such as three pairs of genital papillae, rudimentary chelicerae, and a distinct pharynx—supporting the hypothesis that schizoglyphids are an early-diverging lineage. Although the complete life cycle of *O. maorianus* remains unknown, its nymphal morphology provides valuable clues about the identity of its phoretic stage. The phoretic nymph displays features typically associated with heteromorphic deutonymphs in other Astigmata, such as reduced mouthparts and an attachment organ. However, in contrast to the deutonymph, which serves as the predominant phoretic stage in most Astigmata, the presence of three pairs of genital papillae is strongly indicative of a tritonymphal phoretic stage. If this inference is correct, it would suggest that early Astigmata exhibited greater developmental plasticity, with different lineages exploring alternative phoretic strategies—tritonymphal phoresy in schizoglyphids and deutonymphal phoresy in non-schizoglyphid lineages.

Early Astigmata could have undergone a rapid diversification event, with multiple lineages exhibiting alternative life cycle strategies—such as deutonymphal, tritonymphal, or even adult phoresy. This initial diversification phase would likely have been followed by a convergence toward a dominant strategy as ecological niches became increasingly saturated and competition intensified. Early burst models best explain this kind of diversification pattern, where life history strategies diversify rapidly and subsequently decline due to diversity-dependent processes, including competitive exclusion by lineages with more efficient adaptations [24,25,26,27].

Tritonymphal phoresy, as potentially exemplified by *O. maorianus*, offers a unique strategy that may balance the benefits of phoretic specialization—characteristic of deutonymphal stages—with the advantages of reproductive readiness, typical of adult phoresy. This combination could represent an optimal compromise, reducing trade-offs between dispersal efficiency and developmental timing. Nevertheless, the rarity of tritonymphal phoresy among extant Astigmata raises questions about why this strategy did not persist more broadly. We propose that several factors may have favored the rise of deutonymphal phoresy. These include faster developmental rates and the evolution of progressive paedomorphosis—the retention of juvenile traits in the adult stage—which may have reduced the energetic costs of development by bypassing the production of functionally redundant or metabolically expensive adult features.

Early astigmatid mites likely underwent trait streamlining, including reductions in extensive adult sclerotization (used for predator defense) and a simplification of leg and body setae, many of which may have had overlapping functions. These reductions, coupled with faster development, may have conferred competitive advantages in non-schizoglyphid lineages. While these lineages diversified across a wide range of vertebrate and invertebrate hosts [92], schizoglyphids appear to have retained a more specialized, long-term association with termites [2]—a case of ecological niche conservatism. Termite nests, being relatively stable and long-lived environments, may have buffered schizoglyphids from interspecific competition and supported the persistence of slower developmental rates. In such contexts, the slower-developing life cycle of schizoglyphids could even have provided a local competitive advantage over faster-developing, generalist astigmatid mites. Termite nests also host a diverse assemblage of oribatid mites [98], some of which may specialize in these environments and exploit phoresy during the dispersal of alate termites to new nest sites. While phoretic oribatids have been documented on cockroaches [38]— a general lineage that gave rise to termites [99]—they have not yet been recorded on termites, possibly due to limited large-scale sampling.

Alternatively, the schizoglyphid phoretic stage may be a deutonymph in which the genital papillae develop earlier [17], this would imply that the dominant deutonymphal dispersal strategy evolved and more directly at the origin of Astigmata, with less evolutionary experimentation than currently suspected. However, the morphological evidence from *Oriboglyphus* aligns more strongly with an early burst scenario, in which diverse life cycle strategies emerged rapidly during early astigmatid evolution before converging toward the more efficient deutonymphal phoresy observed in most extant lineages.

This article aims to stimulate further research into phoresy-related metamorphosis and its role in the early evolutionary diversification of Astigmata.

## 5. Conclusions

The evolution of phoresy-related metamorphosis in Astigmata illustrates the interplay between developmental plasticity, ecological opportunity, and selection for dispersal efficiency. Our synthesis suggests that early astigmatid mites evolved a range of ontogenetic pathways for dispersal, including tritonymphal, deutonymphal, and perhaps also adult phoresy, reflecting an initial burst of evolutionary experimentation. This period of diversification likely enabled Astigmata to exploit a broad array of hosts and habitats. Over time, however, competitive pressures and niche saturation appear to have favored the streamlining of life cycles and the emergence of a dominant dispersal strategy based on deutonymphal phoresy. The discovery of *Oriboglyphus maorianus* provides rare insight into this transitional phase, potentially presenting evidence of a tritonymphal phoretic stage. Its morphology suggests that early-diverging lineages may have employed alternative, now largely lost, life cycle strategies—offering a snapshot of evolutionary experimentation before convergence. While tritonymphal phoresy could have offered a functional compromise between dispersal and reproductive readiness, the rise of deutonymphal phoresy was likely associated with accelerated paedomorphosis in this lineage, streamlining energetically costly adult traits and enabling overall faster developmental rates. The persistence of putatively ancestral traits in schizoglyphids, along with their apparent ecological conservatism in termite nests, further indicates the role of ecological context in shaping life history evolution. Whether the phoretic stage in schizoglyphids is truly a tritonymph or a highly derived deutonymph remains an open question—but either scenario has important implications for reconstructing the evolutionary history of this group.

## Figures and Tables

**Table 1:** Leg setation and solenidia of *Oriboglyphus maorianus* gen. et sp. n.

Leg	Tr	Fe	Ge	Ti	Ta
I	*Pr*	*vF*	*cG*, σ	*gT*, *hT*, φ	*aa*, *ba*, *f*, *e*, *d*, *la*, *wa*, *q*, *p*, ω_1_, ω_2_, ω_3_, ε
II	*Pr*	*vF*	*cG*, σ	*gT*, *hT*, φ	*aa*, *f*, *e*, *d*, *la*, *wa*, *q*, *p*, ω_1_, ω_3_
III	*sR*	-	-	*kT*, φ	*d*, *e*, *f*, *r*, *w*, *s*, *q*, *p*
IV	-	-	-	*kT*	*d*, *e*, *f*, *r*, *w*, *s*, *v*, *q*, *p*

## Data Availability

Dataset available on request from the authors.
